# Bio-Inspired Magnetically Controlled Reversibly Actuating Multimaterial Fibers

**DOI:** 10.3390/polym15092233

**Published:** 2023-05-08

**Authors:** Muhammad Farhan, Daniel S. Hartstein, Yvonne Pieper, Marc Behl, Andreas Lendlein, Axel T. Neffe

**Affiliations:** 1 Institute of Active Polymers, Helmholtz-Zentrum Hereon, Kantstr. 55, 14513 Teltow, Germany; muhammad.farhan@hereon.de (M.F.); daniel.hartstein@online.de (D.S.H.); yvonne.pieper@hereon.de (Y.P.); marc-behl@gmx.de (M.B.); andreas.lendlein@gmail.com (A.L.); 2 Institute of Chemistry, University of Potsdam, 14476 Potsdam, Germany

**Keywords:** tendrils, plant inspired movements, remote actuation, magnetic nanocomposite, soft actuators and robotics, shape-memory polymers, multimaterial fibers, inductive heating

## Abstract

Movements in plants, such as the coiling of tendrils in climbing plants, have been studied as inspiration for coiling actuators in robotics. A promising approach to mimic this behavior is the use of multimaterial systems that show different elastic moduli. Here, we report on the development of magnetically controllable/triggerable multimaterial fibers (MMFs) as artificial tendrils, which can reversibly coil and uncoil on stimulation from an alternating magnetic field. These MMFs are based on deformed shape-memory fibers with poly[ethylene-*co*-(vinyl acetate)] (PEVA) as their core and a silicone-based soft elastomeric magnetic nanocomposite shell. The core fiber provides a temperature-dependent expansion/contraction that propagates the coiling of the MMF, while the shell enables inductive heating to actuate the movements in these MMFs. Composites with mNP weight content ≥ 15 wt% were required to achieve heating suitable to initiate movement. The MMFs coil upon application of the magnetic field, in which a degree of coiling *N* = 0.8 ± 0.2 was achieved. Cooling upon switching OFF the magnetic field reversed some of the coiling, giving a reversible change in coiling ∆*n* = 2 ± 0.5. These MMFs allow magnetically controlled remote and reversible actuation in artificial (soft) plant-like tendrils, and are envisioned as fiber actuators in future robotics applications.

## 1. Introduction

Plant tendrils produce fascinating hybrid bending and chiral twisting motion modes, including helical coiling in response to specific external stimuli such as sunlight, humidity, wetting and other atmospheric conditions [1,2]. This coiling has been associated with length and strain mismatch within tendril tissues driven from variations in internal structures, microfibril orientation, rigidness, expansion or swelling behavior [3,4]. Climbing plants use these fibrous structures to obtain support against external mechanical loading such as gusts of wind, or to reach to sunlight or other ecological niches acting as a contracting actuator. Typical tendrils perform three subsequent movements; namely, a “searching” movement, where the untethered end of the young tendril moves autonomously to find support, followed by an “attaching” movement to this support upon contact and lastly a “coiling” movement, which results in forming a spring-like structure, thus shortening the length and consequently pulling the stem closer to the support. To enable biomimetic innovations, special attention has been paid to understanding and replicating the coiling of these plant structures [5,6,7]. Multifunctional materials that allow the incorporation of specific build-in strain mismatch [8,9,10] or the design of self-shaping materials with tunable internal stress that respond to external stimuli [11,12,13,14] have been the strategies to realize similar coiling.

The inspiration from the biological system has led to the development of artificial plant-like movements and devices. The reported multimaterial systems often include composite materials with dissimilar properties of different layers [1,15]. Another strategy to mimic plant coiling is based on liquid crystalline elastomers, where a strong coupling between orientational order and mechanical strain allows spiral- and spring-like deformations when submitted to a stimulus-induced decrease of order [13,16]. In addition to these approaches, single-layer hydrogel sheets with chemically distinct small-scale fiber-like patterns have been reported, which exhibit differential shrinkage and elastic moduli under the application of an external stimulus to give cylindrical and conical helices with specific structural characteristics [16,17]. These approaches not only require complex synthesis strategies, but mostly allow only one-way movements, where the material can take the shape of a contracted, coiled spring upon the application of external stimuli. Most recently, we reported multimaterial fibers based on a shape-memory core fiber (SMCF) and an elastomeric shell, where the reversible movements were reported upon cyclic heating and cooling, i.e., a reversible contracting–expanding spring-like movement [18]. The reversible shape-memory effect in semi-crystalline polymer networks is their ability to actuate between two temporary shapes upon programming and the subsequent stimulation by an external stimulus. This effect is related to melt-induced contraction of the oriented crystallites upon heating and the crystallization-induced elongation of the melted crystals upon cooling in the direction of orientation. In multimaterial fibers, a combined effect of reversible movement of the core and the elastic shell led to reversible coiling instead of linear shape change. In this earlier work, a direct heating/cooling method was used to trigger the movement by putting the specimen in hot and cold water. Such conventional stimuli and the direct contact of the specimen to the stimuli source limit the application of the actuators for advanced robotic applications.

An actuator that can be controlled by magnetic signals eases its deployability as a completely untethered action and offers great potential for applications such as in drug delivery, soft robots and in biomedical engineering. The incorporation of magnetic fillers such as iron oxide nanoparticles into a polymer matrix enables inductive heating through the application of an alternating magnetic field (AMF) remotely. Through the changing magnetic field, a current is induced, called eddy current, while the generated heat is the result of the material’s resistance to the flow of this eddy current by Joule heating [19]. By using a matrix with stimuli responsiveness or actuation in such composites, a magnetic control of the actuation function can be enabled [20,21,22]. The nature of magnetic nanoparticles (mNPs), their concentration and their dispersion profile can influence the inductive heating of polymeric composites in an AMF. Furthermore, the material design (i.e., surface area-to-volume ratio) can critically affect heat transport and in this way the actuation [23,24,25,26]. It has been demonstrated that the heat generated in magneto sensitive shape-memory composites materials by exposure to an AMF results in the melting of a fraction of oriented crystals in the polymer and the partial recovery of the macroscopic shape [27]. The reversible shape change can be observed by switching the magnetic field OFF, which allows the recrystallization of the melted crystals in the direction of orientation.

**Figure 1:**
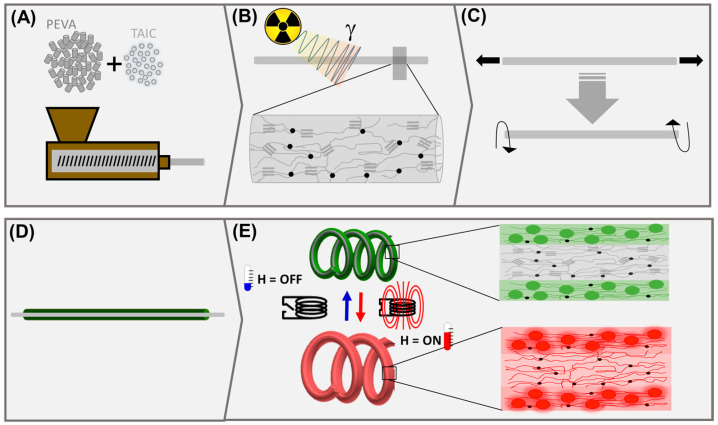
Schematic illustration of the fabrication and actuation of multimaterial fibers via inductive heating. (**A**) The polymer fibers are extruded as a monofilament from a mixture of polymer and crosslinker, i.e., triallyl isocyanurate (TAIC). (**B**) The fibers are crosslinked by gamma irradiation at ambient temperature. The molecular arrangement in the fiber after extrusion and crosslinking is presented with polyethylene crystallites (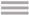), amorphous polymer segments (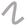) and covalent netpoints (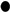). (**C**) The crosslinked fibers are programmed by stretching and twisting. (**D**) MMFs are prepared from a programmed core fiber (by stretching and twisting) and a composite shell comprising mNPs (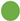) and silicon elastomer presented as amorphous segments (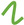). (**E**) The MMF coils upon heating by inductive heating in an AMF and changes reversibly in the number of coils, coil diameter and length on cyclic heating and cooling by switching the field ON and OFF. In the course of inductive heating with magnetic field ON, the heat is generated in mNPs (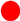) in the shell and transferred to the core, which leads to the melting of PE crystals as indicated by the resulting amorphous system (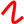), while recrystallization occurs on cooling with the magnetic field OFF. The temperature reached in the composites can be controlled by using a variable current generator as well as by changing the type and amount of mNPs.

The aim of this study was to develop tendril-inspired, fiber-like soft actuators capable of remote controlled reversible coiling movements. For the coiling movement, we relied on the pre-identified concept of multimaterial fibers (MMFs) composed of SMCF and an elastic matrix, i.e., polydimethylsiloxane (PDMS). Here, the outer matrix was designed to enable remote actuation. Therefore, composites comprising silicon and mNPs were developed to serve as a magneto sensitive elastic matrix while crosslinked poly[ethylene-*co*-(vinyl acetate)] (cPEVA) fibers were selected as a core material for the synthesis of remote controlled coiling MMFs. cPEVA was selected for its excellent shape-memory properties. We hypothesized that the shape recovery of the deformed core, which is responsible for the coiling movement, can be triggered via indirect inductive heating of the passive composite matrix in an AMF; see Figure 1. The strategy was to achieve an optimal balance between the mNPs size and the concentration required to achieve sufficient temperature during inductive heating and the overall elasticity and stiffness of the matrix material, so that the core movement was not negatively influenced by the increased stiffness due to incorporated mNPs. Various types of mNPs with different particle sizes were used. Furthermore, the adhesion of the matrix (shell) to the fiber core was important and the delamination of coating during reversible actuation had to be avoided for a reliable multi-cyclic actuation. In this study, only the MMFs capable of reversible coiling movements were targeted, which comprised a pre-stretched and pre-twisted SMCF. The recovery of the deformed core triggered by the inductive heating of a passive matrix can lead to the initial coiling of the MMF, while heating and cooling intervals required for the reversible coiling movement can be achieved by subsequent switching of the magnetic field to OFF and ON.

## 2. Materials and Methods

### 2.1. Materials

Spherical-shaped iron oxide (Fe_3_O_4_) nanoparticles of size 20–30 nm and alpha-Fe_2_O_3_ of 100–200 nm were acquired from SkySpring Nanomaterials, Inc. (Houston, TX, USA). Furthermore, particles of 30–50 nm with Fe_3_O_4_ core and SiO_2_ shell (MagSilica50) were purchased from Fraunhofer ISC (Würzburg, Germany). Polydimethylsiloxane (PDMS) as silicon elastomer base and curing agent (SYLGARD™ 184 Silicone Elastomer Kit), (Dow chemicals, Midland, MI, USA) were used as received. Polytetrafluoroethylene (PTFE) tubes with inner diameters of 1 and 2 mm (VWR International GmbH, Radnor, PA, USA) were used to prepare molds. Poly[ethylene-*co*-(vinyl acetate)] (PEVA) with a vinyl acetate content of 28 wt% (Elvax 260A, DuPont, Wilmington, DE, USA) and triallyl isocyanurate (TAIC) (99%, Sigma–Aldrich, Steinheim, Germany) were used as delivered by the manufacturer.

### 2.2. Methods

Fabrication of Shape-memory Core Fibers (SMCF): SMCF were prepared from a mixture of 99 wt% of PEVA and 1 wt% of TAIC. At first, PEVA granulates and TAIC were mixed in a twin-screw extruder (Euro Prism Lab, Thermo Fisher Scientific, Waltham, MA, USA) at 110 °C and 50 rpm. In the next step, the blend granulates (PEVA + TAIC) were extruded using a single screw extruder (Extrudex, Mühlacker, Germany) to obtain fibers. After fabrication by extrusion, the fibers were cross-linked by a gamma beam of 165 kGy in a post-extrusion process.

Fabrication of Multimaterial Fibers (MMFs): Fabrication can be divided into three steps of preparation of the core as well as shell material and the formation of MMFs.

Core material: Shape-memory core fibers were used in the programmed shape obtained by stretching and twisting. At first, cPEVA fibers with a diameter of 0.4 mm and 15 cm length were relaxed in hot water (≥80 °C) to remove any strain from extrusion process. Then, they were deformed by stretching by 100% followed by a second deformation step by twisting. The number of twists was 30 twists per 12 cm core material length after stretching.

Shell material: Silicone elastomer base and curing agent were mixed in 13:1 ratio with 5 to 20 wt% mNP manually with a glass stirring rod and degassed under vacuum for half an hour to remove air bubbles.

Formation of MMFs: A process similar to the one defined in [18] was adopted. Two PTFE tubes with 1 and 2 mm diameter were used as molds. The inner molding tube with 1 mm diameter was cut through the middle from one end to the other end but leaving 5 mm, such that the two halves were still attached together. A second tube with a larger diameter served as a casing for the inner mold to avoid the flow of the solution out of the mold. The outer casing tube was cut on one side along the length for easier insertion and removal of the inner mold. The core material was then inserted into the inner molding tube. The mold was then closed on one side using Teflon tape, while the other side was left open until the insertion of the shell material mixture (silicon-curing agent-mNP). Filled molds were finally inserted into a glass tube and were placed in a water bath at 50 or 60 °C overnight for curing.

Mechanical Properties of the composite samples were investigated on a Zwick BZ2.5/TN1S (Zwick Roell, Ulm, Germany) while experimental data were captured with testXpert II (Zwick Roell, Ulm, Germany). The tests were conducted at room temperature with a strain rate of 5 mm·min^−1^, and elastic modulus (*E*) and elongation at break (*ε*_break_) were obtained and analyzed.

Transmission Electron Microscopy (TEM) was used to investigate size, shape and agglomeration of all commercially acquired magnetic nanoparticles. The particles were dispersed in isopropanol, ultrasonicated for 15 min and drop-casted on a lacey-C covered Cu-grid of 400 mesh. The samples were investigated at room temperature using a single tilt holder in bright field mode on Thermo Scientific™ Talos™ F200X (Thermo Fisher Scientific, Waltham, MA, USA) equipped with a Ceta 16M CMOS camera and X-FEG electron source operated at 200 kV, and images were taken with a magnification between 34,000× and 1,050,000×.

Core-shell geometry and distribution of mNPs in the shell composite: A light microscope Ax10 (Carl Zeiss Microscopy GmbH, Oberkochen, Germany) equipped with imager A1m and a digital camera (AxioCam 506 color) was used to investigate the cross-section of the MMFs. The samples were obtained by simple blade cutting at room temperature from various sections of the MMFs, such as from the sides and the center part, while the images were taken with 10×, 20× and 50× magnification under transmission and reflection light.

Inductive Heating: At first, the heating of the composite shell material was investigated from the solution-casted film using a rectangular test specimen (20 × 10 × 1 mm^3^). The samples were positioned in an AMF at a frequency of f = 258 kHz. Samples were fixated with a split wooden stick fully inside the coil. The equipment consisted of a high-frequency generator (TIG 5/300; Huettinger Electronic, Freiburg, Germany) and a copper coil (six loops, diameter 4 cm, height 4.5 cm). The magnetic field strength (*H*) could be adjusted between 7 and 30 kA·m^−1^ by variation of the power output of the generator. A similar *H* = 30 kA·m^−1^ (37.55 mT) was used for the investigation. It was ensured that all samples had similar proportions and were positioned exactly the same for comparable results. The multimaterial fibers containing a shape-memory core fiber were also investigated for their heating. These MMFs were fixed on the top end of the coil via a wooden stick since the expected coiling would shorten the length and pull the part hanging outside the coil inside the coil, consequently exposing the full length of MMF to AMF. Samples were heated, cooled and heated again multiple times to ensure reproducibility. The surface temperatures for composite shell material as well as for MMFs were recorded with an infrared video camera VarioCAM^®^ HiRes (InfraTec GmbH, Dresden, Germany) and analyzed with IRBIS 2 (Infratec GmbH, Dresden, Germany).

Coiling and reversible actuation investigation: The magnetically triggered reversible actuation of multimaterial fibers was investigated by holding the MMFs inside a magnetic coil, while the AMF with a strength of *H* = 30 kA·m^−1^ (37.55 mT) was switched ON and OFF reversibly to induce inductive heating followed by cooling in each cycle. The initial length of the MMFs was measured before placing them inside the coil. In the next step, the magnetic field was switched ON and the number of coils (*n*_heat_) was counted after an equilibrium time of 2 min for uniform heating. Afterwards, the field was switched OFF and the number of coils was counted again (*n*_cool_) after an equilibrium time of 2 min. This cycle was repeated 3 times per tendril. At the end of the experiment, the length of the (coiled) MMF was measured. The degree of coiling (*N*) can be estimated as the number of coils per unit length of the fiber (*N* = *n/L*). Here, ‘*L*’ is the initial length (*L*_initial_) of the MMF, while ‘*n*’ is the number of coils on heating or cooling (*n*_heat_ or *n*_cool_) by switching the magnetic field ON and OFF. Furthermore, the change in number of coils (∆*n*) during magnetic heating and cooling cycles was calculated as (∆*n* = *n*_heat_ − *n*_cool_).

Statistics: The presented data in this study are average values with respective standard deviations (STD) based on experiments conducted at least five times, unless stated otherwise. Mechanical tests were performed for at least five samples, and average values of elastic modulus and elongation at break were calculated along with STD. The geometry of the multimaterial fibers as well as distribution mNP were analyzed from two to three samples. At least five samples were analyzed for investigating the actuation behavior from each multimaterial fiber type, and average values as well as STD were calculated.

## 3. Results and Discussion

**Figure 2:**
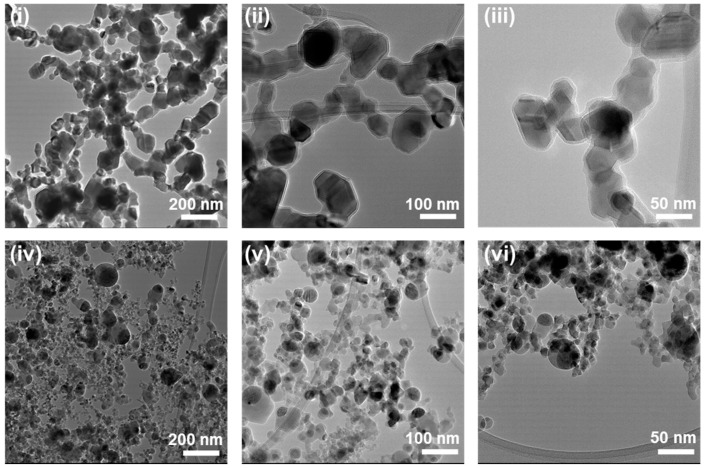
Transmission electron microscopic investigation of the spherical SiO_2_-coated Fe_3_O_4_ mNP (**i**–**iii**) and non-coated spherical Fe_3_O_4_ mNP (**iv**–**vi**).

The commercially obtained iron oxide (Fe_3_O_4_) mNPs were analyzed by transmission electron microscopy (TEM) for their size variations. In general, the obtained particle size was within the range provided by the manufacturer; however, a few outliers of the noted sizes could be found, as shown in Figure 2. Agglomeration could be observed in all nanoparticles, which was expected, as noted in the literature [28]. The size of the non-coated spherical Fe_3_O_4_ mNPs (Sp20) ranged between 20 and 30 nm, with a very low population having a size of ~50 nm. The ‘Sp’ in the abbreviation refers to spherical, while the number indicates the particle size in nm. In some places, large dark agglomerations can be seen, which complicated the image analysis and size determination. The TEM investigations of SiO_2_-coated mNPs (Sp30) confirmed the average particle size to be between 30 and 50 nm. Again, a very small number of particles were observed with sizes up to ~100 nm. The protective SiO_2_ shell was also visible, with a thickness of around 5 nm.

The blurriness of all TEM images can be explained due to the nature of the samples. Heavy iron oxide molecules bend the electron beam, creating a certain inaccuracy, resulting in blurriness [29]. Furthermore, similar to a light microscope, an image is created of a 3D sample, focusing only on one depth inside it. Particles under or above this level are blurry, but can still be visible. Additionally, the sample can move while imaging due to the size of the sample and the reaction between the sample and the electron beam.

**Figure 3:**
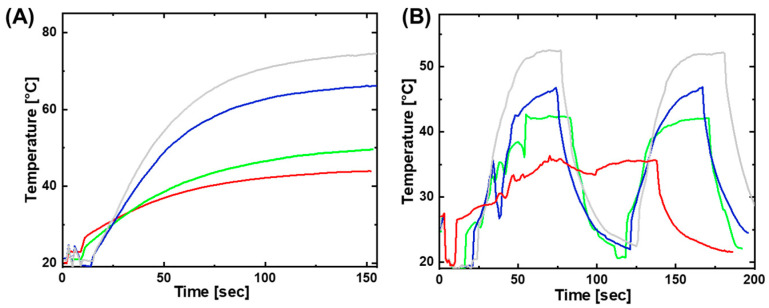
Heating profile of PDMS-mNP composite upon stimulation in alternating magnetic field; PDMS-SP20-15 (red), PDMS-Sp20-20 (green), PDMS-Sp30-15 (blue), and PDMS-Sp30-20 (light grey). (**B**) Heating and cooling profiles of MMFs containing SMCF and a PDMS-mNP as shell, with the same color code as in (**A**).

The magnetic heating capability of the three studied mNP types was investigated in PDMS-mNP composites with mNP weight content between 5 and 20 wt%. The nomenclature for the composites used is PDMS-SpXX-YY, with XX referring to the particle size in nm and YY to the particle loading in wt%. The 2D composite films were evaluated for their magnetic heating without any active shape-memory core/layer. The aim was to achieve heating to a temperature between 55 and 65 °C, which is within the broad melting range of the cPEVA to be used as a core for magnetically triggered MMFs [21]. This temperature is necessary to trigger coiling in such MMFs which contain temperature sensitive SMCF. These preliminary investigations showed that an mNP weight content of at least 15 wt% was necessary to reach the desired temperature. Therefore, composites with less than 15 wt% mNP were considered out of the scope of this study, and their results were excluded. The heating profiles of the composites with 15 and 20 wt% mNP are shown in Figure 3A, which corresponded to the heating of rectangular samples 1 × 2 cm with a thickness between 1 and 2 mm. The results show that the heating of Sp30 was better when compared with Sp20, as a higher temperature could be reached with the same magnetic field strength *H* = 30 kA·m^−1^. The Sp100 composites did not heat up to the required temperatures (see Supporting Information Figure S1), and were therefore not further investigated. An increase in the wt% of mNPs increased the possible inductive heating of a composite. This was due to the fact that more mNPs increased the possible saturation magnetization and therefore heat creation. A similar behavior occurred when increasing the size of the mNPs. The heating profile formed a selection criteria for the PDMS-mNP composite material, according to which formulations with 15 and 20 wt% mNP were selected as a shell material for MMFs covering the SMCF and responsible for magnetically triggered coiling. 

Afterwards, the MMFs were synthesized with the SMCF and PDMS-mNP as shell, and the magnetic heating of these MMFs was investigated and compared with that of composites. The diameter of the SMCF was 0.4 ± 0.1 mm, while the diameter of the MMFs was 1 mm and the influence of the changes of the diameter of core and shell on the properties of MMFs was not investigated in this study. The nomenclature for the MMFs is MMF-SpXX-YY, with XX referring to the particle size in nm and YY to the particle loading in wt%. The results are shown in Figure 3B, where comparable heating profiles can be obtained for MMFs as for PDMS-mNP composites (without SMCF), though somewhat lower temperatures were reached, likely because in the MMFs some parts did not contain mNPs.

**Figure 4:**
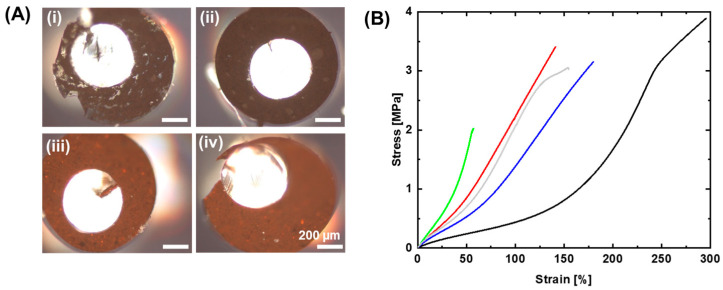
(**A**) The POM images of MMFs: (**i**) MMF-Sp20-15, (**ii**) MMF-Sp20-20, (**iii**) MMF-Sp30-15 and (**iv**) MMF-Sp30-20. (**B**) Stress–strain curves of PDMS-mNP composite fibers (without SMCF): PDMS-Sp20-15 (red), PDMS-Sp20-20 (green), PDMS-Sp30-15 (blue) and PDMS-Sp30-20 (light grey) in comparison to PDMS (black).

The distribution of mNPs in the PDMS-mNP shell was investigated by polarized optical microscopy (POM) of the cross-sections of MMFs, and the results are presented in Figure 4A. These images show an even distribution of mNPs through the entire MMFs. The more-or-less even distribution of the mNPs guarantees optimal thermal induction and a uniform mechanical behavior of the composite throughout the MMF. These images also show the location of the inner fiber, which is not always centered [18]. This can have an influence on the coiling behavior of these tendrils. Since the location of the fiber cannot be fully controlled, a variation in similar synthesized MMFs is to be expected.

The mechanical properties of the PDMS, PDMS-mNP composite fibers and of MMFs were investigated with tensile tests at ambient temperature and the results are shown in Figure 4B and Table 1. A lower modulus *E* = 0.68 ± 0.01 MPa was observed for pure PDMS, while the results of PDMS-mNP composite fibers revealed an increase in *E* with an increase in the weight content of mNPs in these composite fibers. The modulus of PDMS-Sp20 increased from 1.85 to 2.28 MPa with an increase in mNP content from 15 to 20 wt%, while the elongation at break (*ε*_b_) decreased from 120 ± 26 to 60 ± 23%. Similarly, the *E* of PDMS-Sp30 increased from 1.58 to 2.02 MPa and *ε*_b_ decreased from 180 ± 39 to 158 ± 61% with a similar increase in mNP wt%. This is due to the fact that implementing a harder material into a softer one increases its stiffness, and therefore the Young’s modulus increases while its deformability is reduced. The stress–strain curves in Figure 4B indicate that an increase in the size of the mNPs reduces its influence on the Young’s modulus. A reason behind this could be that bigger particle sizes (with the same wt% in a composite) mean fewer stiff particles inside the composite, leaving a denser network of the elastic polymer. The mNPs reside in the crosslinked PDMS and interfere with its elasticity. The influence of the particles on the crosslink density of the network results in a reduction in elongation at break. In fact, it has been shown in the literature [30] that the mechanical properties of a composite are especially sensitive when particle sizes below a critical size, depending on the matrix, the particles and the matrix/particle adhesion, are used. A decrease in particle size below this threshold is then associated with, e.g., an increase in Young’s modulus.

In addition to the PDMS-mNP composite fibers, mechanical properties of MMFs were investigated. However, the results did not follow an observable trend, and fluctuated a lot. During testing, it was observable that the composite shell ripped apart, delaminating the core, and SMCF continued to stretch at the breakage of the shell. Therefore, it was concluded that tensile testing is not viable for a mechanical investigation of the MMFs and the results were excluded from this study. Overall, the influence of the composite shell was negligible and the SMCF dominated the mechanical behavior of these MMFs.

**Figure 5:**
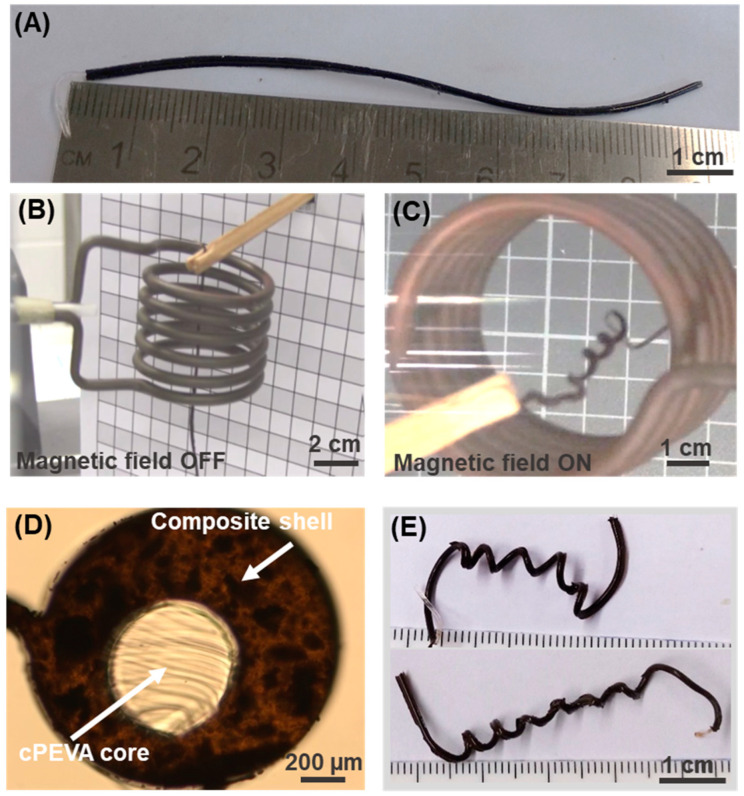
(**A**) Image of the MMF after synthesis and before actuation. (**B**) MMF held in the magnetic coil before its actuation and with magnetic field turned OFF. (**C**) MMF in the magnetic coil after heating by turning the magnetic field ON, and consequent coiling. (**D**) Cross-section of coiled MMF. (**E**) Macroscopic images of coiled MMFs after their actuation by magnetically triggered heating. All images in this figure are from MMF-Sp30-20. Brightness of images was increased for better readability.

The magnetically triggered MMFs were prepared containing a cPEVA core that was already programmed by stretching and twisting, so that it would coil upon the application of a magnetic field and resulting inductive heating, as is demonstrated in Video S1 and Figure 5. The structural changes in the SMCF after deformation and before embedding into the shell have been shown earlier via microscopic investigation [18]. Table 1 gives the number of coils observed in the MMF on heating as well as cooling as an average of five cycles. This number of coils can be used to calculate the degree of coiling (*N*), which is the number of coils (*n*_heat_ or *n*_cool_) per unit length (*L*_initial_) of the MMF. The MMF tended to coil instantly upon heating by induction, resulting in a high *N* = 0.8 ± 0.2 for the MMF-Sp30, while a lower degree of coiling of *N* = 0.6 ± 0.15 for the MMF-Sp20 was observed. Since the actively moving SMCF had the same deformation for both MMF types, this difference in *N* might be due to the lower temperature achieved for MMF-Sp20 upon magnetic heating at a similar magnetic field strength. The cooling to room temperature, on the other hand (by switching OFF the magnetic field), led to the reversing of the degree of coiling and resulted in a smaller number of coils in the respective MMF. However, the difference in the number of coils on heating and cooling ∆*n* = 2 ± 0.5 (averaged over all samples) was not that high. In addition, these magnetically controlled MMFs did not form a compressed spring-like conformation upon cooling, as was observed for the thermally driven MMF actuators [18]. This might be attributed to the higher stiffness of the PDMS-mNP shell in such MMFs compared to that of the softer PDMS shell in thermally driven MMF actuators. The reversible coiling actuation in magnetically controlled MMF can be observed in Video S1 for four heating-cooling cycles. The reversible actuation process can be repeated several times, and a similar reversible change in the length was obtained in subsequent cycles. The MMFs behaved similarly; however, the coiling behavior of the MMFs can be changed by changing the deformation of the core, such as by changing the number of twists or by changing the programming strain. Due to the restricted view on the MMF inside the magnetic coil, the change in length (∆*L*) upon heating and cooling could not be quantified accurately. However, the observed change in length was much smaller than in the thermally controlled MMF actuators, which again could be attributed to the stiffness of the shell. The initial length of MMF and the final length indicated in Table 1 are lengths before and after the end of the experiment and the removal of the samples from the magnetic coil, and must not be mistaken for the estimation of ∆*L*.

The reversible coiling movements can be explained as a combined phenomenon of core–shell MMFs where the core as an active component is mainly responsible for the movement. The core is deformed by stretching and twisting, which when firmly adhered to the elastomeric shell leads to coiling or reversible actuation upon a change in temperature by inductive heating. Shape-memory fiber actuators without an elastomeric shell behave differently; a fiber programmed by stretching or twisting would either contract or untwist on heating, with a reciprocal movement on cooling. Therefore, combined programming by stretching and twisting implies contraction and untwisting on heating, while elongating and twisting on cooling. The reversible coiling movement is a result of the presented core–shell MMFs, where the active SMCF is the driving force, while the elastomeric shell hinders the individual movement of the core, resulting in a coiling movement instead. The change in temperature leads to the melting of the crystallites or their recrystallization in the SMCF. This happens in a partially directed orientation due to the residual tension of the core being restricted from total relaxation by the attached shell. Such nanoscale changes through crystallization lead, then, to macroscopic movement, which in this case resulted in reversible coiling. These MMFs can be actuated as long as the core and shell adhere to one another and are not delaminated, which would result in total relaxation and no more directed crystallization. Plant tendrils are mostly support elements and are active under external stress and tension. In the artificial MMFs, such forces might not be possible with a single MMF; however, such limitations can be overcome by an assembly of multiple MMFs.

## 4. Conclusions

In this study, we have reported magnetically controlled plant-tendril-inspired coiling actuators. The design principles for the desired coiling movements are based on plant fibril structures. Here, suitable formulations from PDMS and mNP for a composite shell enabling inductive heating were developed. Two types of commercially available mNP, non-coated Fe_3_O_4_ (Sp20) with a particle size ~20–30 nm and SiO_2_-coated mNP (Sp30) of ~30–50 nm, were used. The initial results showed that an mNP weight content of ≥15 wt% was required to achieve heating to a temperature ≥40 °C. The heating of composites containing Sp30 mNPs was more effective when compared with composites containing Sp20 or Sp100 mNPs, as a higher temperature could be reached with the same magnetic field strength of *H* = 30 kA·m^−1^. Tensile tests of composite fibers revealed an increase in *E* with an increase in the weight content of mNPs in these composites. The modulus of PDMS- Sp20 increased from 1.85 to 2.28 MPa with a change in the mNP content from 15 to 20 wt%, while the elongation at break (*ε*_b_) decreased from 120 ± 26 to 60 ± 23%. Contrary to possible assumptions, an increase in the size of mNPs reduced the impact on the Young’s modulus while increasing the heatability of the composite. Based on these results, MMFs with 15 and 20 wt% mNP were prepared from the Sp20 and Sp30 mNP types and were investigated for their coiling behaviors. The POM investigation confirmed the uniform distribution of mNP in the PDMS-mNP shell. A high degree of coiling of *N* = 0.8 ± 0.2 was observed for the MMF-Sp30 on inductive heating in an alternating magnetic field. The cooling of the MMF by switching OFF the magnetic field reversed the coiling to some extent, and a reversible change in coiling ∆*n* = 2 ± 0.5 was observed on heating and cooling. These MMFs allowed magnetically controlled remote actuation in artificial (soft) plant-like tendrils and are envisioned as fiber actuators in future robotic applications.

## Figures and Tables

**Table 1:** Mechanical properties of PDMS and PDM-mNP composite fibers along with coiling behaviors of various MMFs.

Sample	*E* ^a)^ [MPa]	*ε*_b_ ^b)^ [%]	*L*_initial_ ^c)^ [cm]	*L*_final_ ^d)^ [cm]	*n*_heat_ ^e)^ [#]	*n*_cool_ ^f)^ [#]	∆*n* ^g)^ [#]
PDMS-Sp20-15	1.85 ± 0.01	120 ± 26	10.2 ± 1.0	4.8 ± 1.0	6 ± 2.2	3.9 ± 2.0	2.1 ± 0.6
PDMS-Sp20-20	2.28 ± 0.01	60 ± 23	10.0 ± 0.3	4.4 ± 1.2	5.5 ± 1.5	3.6 ± 1.3	1.9 ± 0.5
PDMS-Sp30-15	1.58 ± 0.02	180 ± 39	9.4 ± 0.5	4.1 ± 0.2	8.9 ± 1.8	6.6 ± 1.8	2.3 ± 0.4
PDMS-Sp30-20	2.02 ± 0.01	158 ± 61	9.8 ± 0.7	4.7 ± 0.4	8.5 ± 1.9	7.0 ± 1.9	1.5 ± 0.3
PDMS	0.68 ± 0.01	188 ± 107	-	-	-	-	-

^a)^ E: Young’s modulus of PDMS and PDMS-mNP composite fibers. ^b)^ *ε*_b_: Elongation at break of PDMS and PDMS-mNP composite fibers. ^c)^ *L*_initial_: Length of MMF before actuation. ^d)^ *L*_final_: Length of MMF after actuation. ^e)^ *n*_heat_: Number of coils upon magnetic heating. ^f)^ *n*_cool_: Number of coils after cooling. ^g)^ ∆*n*: Change in number of coils during magnetic heating and cooling cycles.

## Data Availability

The data presented in this study are openly available in zenodo at DOI: 10.5281/zenodo.7810297.

## Supplementary Materials

The following supporting information can be downloaded at: https://www.mdpi.com/article/10.3390/polym15092233/s1, Figure S1: Heating profile of PDMS-mNP composite containing an alpha-Fe_2_O_3_ type mNP of 100–200 nm size: PDMS-SP100-5 (red), PDMS-Sp100-15 (green), and PDMS-Sp100-20 (blue). Supporting Video S1: The reversible coiling actuation of MMF-Sp30-20 in four heating–cooling cycles.

## References

[1] Wang W., Li C., Cho M., Ahn S.H. (2018). Soft Tendril-Inspired Grippers: Shape Morphing of Programmable Polymer-Paper Bilayer Composites. ACS Appl. Mater. Interfaces.

[2] Wang M., Lin B.-P., Yang H. (2016). A plant tendril mimic soft actuator with phototunable bending and chiral twisting motion modes. Nat. Commun..

[3] Armon S., Efrati E., Kupferman R., Sharon E. (2011). Geometry and Mechanics in the Opening of Chiral Seed Pods. Science.

[4] Gerbode S.J., Puzey J.R., McCormick A.G., Mahadevan L. (2012). How the Cucumber Tendril Coils and Overwinds. Science.

[5] Wong S.K., Chen K.C. (2016). A Procedural Approach to Modelling Virtual Climbing Plants With Tendrils. Comput. Graph. Forum.

[6] Flynn C.M., Puzey J.R. (2017). A Comparative Study of the Biomechanics of Coiling Tendrils. Integr. Comp. Biol..

[7] Godinho M.H., Canejo J.P., Pinto L.F.V., Borges J.P., Teixeira P.I.C. (2009). How to mimic the shapes of plant tendrils on the nano and microscale: Spirals and helices of electrospun liquid crystalline cellulose derivatives. Soft Matter.

[8] Cheng Y., Wang R.R., Chan K.H., Lu X., Sun J., Ho G.W. (2018). A Biomimetic Conductive Tendril for Ultrastretchable and Integratable Electronics, Muscles, and Sensors. Acs Nano.

[9] Zhao Y., Miao X., Lin J., Li X., Bian F., Wang J., Zhang X., Yue B. (2017). Coiled Plant Tendril Bioinspired Fabrication of Helical Porous Microfibers for Crude Oil Cleanup. Glob. Chall..

[10] Chen P.N., Xu Y.F., He S.S., Sun X.M., Pan S.W., Deng J., Chen D.Y., Peng H.S. (2015). Hierarchically arranged helical fibre actuators driven by solvents and vapours. Nat. Nanotechnol..

[11] Hines L., Petersen K.H., Lum G.Z., Sitti M. (2017). Soft Actuators for Small-Scale Robotics. Adv. Mater..

[12] de Haan L.T., Verjans J.M.N., Broer D.J., Bastiaansen C.W.M., Schenning A.P.H.J. (2014). Humidity-Responsive Liquid Crystalline Polymer Actuators with an Asymmetry in the Molecular Trigger That Bend, Fold, and Curl. J. Am. Chem. Soc..

[13] Iamsaard S., Asshoff S.J., Matt B., Kudernac T., Cornelissen J.J.L.M., Fletcher S.P., Katsonis N. (2014). Conversion of light into macroscopic helical motion. Nat. Chem..

[14] Lendlein A. (2018). Fabrication of reprogrammable shape-memory polymer actuators for robotics. Sci. Robot..

[15] Boothby J.M., Ware T.H. (2017). Dual-responsive, shape-switching bilayers enabled by liquid crystal elastomers. Soft Matter.

[16] Therien-Aubin H., Wu Z.L., Nie Z.H., Kumacheva E. (2013). Multiple Shape Transformations of Composite Hydrogel Sheets. J. Am. Chem. Soc..

[17] Wu Z.L., Moshe M., Greener J., Therien-Aubin H., Nie Z., Sharon E., Kumacheva E. (2013). Three-dimensional shape transformations of hydrogel sheets induced by small-scale modulation of internal stresses. Nat. Commun..

[18] Farhan M., Klimm F., Thielen M., Rešetič A., Bastola A.K., Behl M., Lendlein A., Speck T. (2023). Artificial tendrils mimicking plant movements by mismatching modulus and strain in core and shell. Adv. Mater..

[19] Xiang Z.Y., Ducharne B., Della Schiava N., Capsal J.F., Cottinet P.J., Coativy G., Lermusiaux P., Le M.Q. (2019). Induction heating-based low-frequency alternating magnetic field: High potential of ferromagnetic composites for medical applications. Mater. Des..

[20] Enriquez E., de Frutos J., Fernandez J.F., de la Rubia M.A. (2014). Conductive coatings with low carbon-black content by adding carbon nanofibers. Compos. Sci. Technol..

[21] Farhan M., Chaudhary D., Nochel U., Behl M., Kratz K., Lendlein A. (2021). Electrical Actuation of Coated and Composite Fibers Based on Poly[ethylene-co-(vinyl acetate)]. Macromol. Mater. Eng..

[22] Park J., Yoo J.W., Seo H.W., Lee Y., Suhr J., Moon H., Koo J.C., Choi H.R., Hunt R., Kim K.J. (2017). Electrically controllable twisted-coiled artificial muscle actuators using surface-modified polyester fibers. Smart Mater. Struct..

[23] Jang J.T., Lee J., Seon J., Ju E., Kim M., Kim Y.I., Kim M.G., Takemura Y., Arbab A.S., Kang K.W. (2018). Giant Magnetic Heat Induction of Magnesium-Doped gamma-Fe_2_O_3_ Superparamagnetic Nanoparticles for Completely Killing Tumors. Adv. Mater..

[24] Zhang F.H., Zhang Z.C., Luo C.J., Lin I.T., Liu Y.J., Leng J.S., Smoukov S.K. (2015). Remote, fast actuation of programmable multiple shape memory composites by magnetic fields. J. Mater Chem. C.

[25] Heuchel M., Razzaq M.Y., Kratz K., Behl M., Lendlein A. (2015). Modeling the heat transfer in magneto-sensitive shape-memory polymer nanocomposites with dynamically changing surface area to volume ratios. Polymer.

[26] Mohr R., Kratz K., Weigel T., Lucka-Gabor M., Moneke M., Lendlein A. (2006). Initiation of shape-memory effect by inductive heating of magnetic nanoparticles in thermoplastic polymers. Proc. Natl. Acad. Sci. USA.

[27] Razzaq M.Y., Behl M., Heuchel M., Lendlein A. (2020). Matching Magnetic Heating and Thermal Actuation for Sequential Coupling in Hybrid Composites by Design. Macromol. Rapid Commun..

[28] Franks G.V., Lange F.E. (1996). Plastic-to-brittle transition of saturated, alumina powder compacts. J. Am. Ceram. Soc..

[29] Baumgartel C., Smith R.T., Maher S. (2020). Accurately predicting electron beam deflections in fringing fields of a solenoid. Sci Rep..

[30] Fu S.Y., Feng X.Q., Lauke B., Mai Y.W. (2008). Effects of particle size, particle/matrix interface adhesion and particle loading on mechanical properties of particulate-polymer composites. Compos. Part B Eng..

