# Supplementary material for: Bio-Inspired Magnetically Controlled Reversibly Actuating Multimaterial Fibers
Source: Polymers (Basel). 2023 May 8;15(9):2233. doi: 10.3390/polym15092233 (PMC10181395; doi:10.3390/polym15092233)
Supplement: Supplementary file 1 [file polymers-15-02233-s001.zip › 230227_Supporting Information.pdf]

# Supporting Information to

## Bio-inspired magnetically controlled reversibly actuating multimaterial fibers

Muhammad Farhan[1], Daniel S. Hartstein[1], Yvonne Pieper[1], Marc Behl[1], Andreas Lendlein[1,2], Axel T. Neffe[1]

[1]Institute of Active Polymers, Helmholtz-Zentrum Hereon, Kantstr. 55, 14513 Teltow, Germany

[2]Institute of Chemistry, University of Potsdam, 14469 Potsdam, Germany

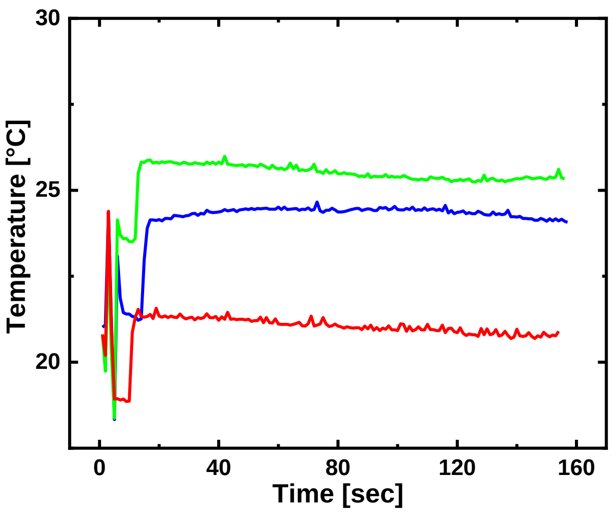


**Figure S1.** Heating profile of PDMS-mNP composite containing alpha-$Fe_2O_3$ type mNP of 100-200 nm size: PDMS-SP100-5 (red), PDMS-Sp100-15 (green), and PDMS-Sp100-20 (blue).
